# Supplementary material for: Energy flows reveal declining ecosystem functions by animals across Africa
Source: Nature. 2025 Oct 29;649(8095):104–12. doi: 10.1038/s41586-025-09660-1 (PMC12727517; doi:10.1038/s41586-025-09660-1)
Supplement: Supplementary file 1 — Supplementary Discussion, Supplementary Fig. 1, Supplementary Tables 1–4 and a descriptive overview of the code used in the analysis. [file 41586_2025_9660_MOESM1_ESM.docx]

**Energy flows reveal declining ecosystem functions by animals across Africa**

**Supplementary information**

Loft et al.

**Supplementary Information Guide**

This paper includes three sets of supplementary materials.

First, the Supplementary Materials document includes (i) supplementary discussion on flat versus variable population densities, and on the relationship between HANPP and the energetic intactness of animal-mediated functions. It also includes (ii) supplementary figure 1, which shows the relationship between HANPP and the intactness of energy flows through birds and mammals, and (iii) tables S1-S4, which present the results of a sensitivity test and data underlying the energy consumption analyses. Finally, it includes (iv) a descriptive overview of the code used in the analysis.

Second, the Supplementary Data 1 presents the full quantitative results of current and historical energy flows through functional groups and guilds, as well as the 95% confidence interval values for these results. These results are presented in the main text within figures.

Third, the Supplementary Data 2 presents data on functional traits, population density, and annual energy consumption for each of the ~3000 bird and mammal species included in the analysis.

**Supplementary Discussion**

*Flat versus variable population densities*

To calculate the energy flows through African birds and mammals, we used a single, range-wide or “flat” estimate of each species’ population density. In reality, intra-species population densities vary across species’ ranges, according to environmental variables: they are not “flat” but “variable”. We used flat densities, because variable densities are only available for a few species. We assumed that our simplifying assumption would not substantially alter our results, hypothesizing that intra-species variation in population densities would even out when summing energy flows across tens, hundreds, or thousands of species.

To test this assumption, we calculated the declines in energy flows through the 92 large herbivore species for which spatially variable population densities are available over the whole of sub-Saharan Africa^1^. We did these calculations using both the spatially explicit variable densities provided by Hempson et al., and using flat densities calculated by averaging the spatially-explicit densities, and then applying average values across the whole of each species’ range. We calculated the difference between (i) the total range-wide energy flows through the guild; (ii) the range-wide energy flows through each individual species, and (iii) the guild-wide energy flows through our four major land uses and three major biomes. The 92 analyzed large herbivore species account for over one quarter the total energy flow through mammals, and are therefore a meaningful proxy for the larger set of species assessed in the main analysis.

We found that assuming flat densities did not meaningfully change the decline in energy flow across any of these categories, except for a few individual species (**Table S1**). The total, range-wide energy flow through large herbivores was 0.8% more intact when calculated using flat densities than using variable densities (25.75% versus 24.96%). In no land use or biome was the difference in energy flows greater than 1%. In addition, the difference in intactness was less than 1% for 72 of the 92 assessed species, with the largest differences apparent for sable antelope (*Hippotragus equinus,* 4.9%*)*, impala (*Aepyceros melampus*, 4.8%) and steenbok (*Raphicerus campestris,* 4.3%). These results indicate that assuming flat densities does not meaningfully change the results of the study, at least for the important large herbivore guild. The results further suggest that although many landscapes have been highly transformed by humans, these transformed landscapes do not correlate with regions that feature exceptionally high or low population densities across a wide suite of species. In other words, intra-species population densities do not vary consistently across species in a way that correlates with highly transformed or protected landscapes. We therefore conclude that using flat densities does not substantially change the key results of our studies.

**Table S1**. Differences in energy flows through 92 large herbivore species calculated using flat range-wide average population densities versus variable, spatially explicit population densities. For the calculations using flat density, each species was assumed to occur in it range-wide mean population density across its entire range. For the variable population densities, we used the spatially explicit estimates in Hempson et al. (*Science,* 2014); these densities varied across the range of each species according to environmental variables (e.g. climate, soil, biome).

| **Group** | Intactness –flat densities | Intactness – variable densities | Difference in intactness |
| --- | --- | --- | --- |
| Total energy flow (large herbivores) | 25.75% | 24.96% | 0.78% |
| Median difference in energy flow for 92 large herbivores species | 32.94% | 32.88% | 0.07% |
| **Land Use** |  |  |  |
| Settlements | 3.68% | 3.68% | 0.00% |
| Croplands | 5.87% | 5.87% | 0.00% |
| Untransformed lands | 26.90% | 26.20% | 0.70% |
| Protected lands | 58.90% | 58.90% | 0.00% |
| **Biome** |  |  |  |
| Forest | 29.99% | 29.15% | 0.84% |
| Grassy | 24.60% | 24.25% | 0.35% |
| Arid | 27.78% | 28.39% | -0.60% |

*Relationship between HANPP and the energetic intactness of animal-mediated functions*

Human-appropriated net primary productivity (HANPP) has recently been proposed as suitable metric to use for determining a planetary boundary for biosphere integrity^2^, replacing the previous metric, biodiversity intactness (BII). We suggest that using HANPP as a solitary metric for biosphere integrity poorly captures changes to the animal component of the biosphere. HANPP estimates the amount of potential plant energy appropriated by humans through agricultural harvests and land use change^3^. A proportionate HANPP value (between 0% and 100%) can be calculated by dividing the absolute HANPP in a cell by the absolute potential NPP of a cell. HANPP is a reasonable proxy for how much humans have impacted the plant component of the biosphere, given that HANPP measure the proportion of plant energy that humans have appropriated. In contrast, HANPP is unlikely to accurately capture change to the integrity of the animal component of the biosphere, for three reasons. First, populations of different animal species and their associated ecosystem functions decline asynchronously in response to different kinds of land use change^4^. For example, rising HANPP in rangelands, where vegetation is consumed by livestock, may simultaneously benefit species adapted to open grassy vegetation, and harm species adapted to closed, woody vegetation^5^. Second, a key driver of change to animal populations—overharvesting—is uncorrelated or very weakly correlated with changes to NPP. Ecosystems with intact vegetation, and thus low HANPP, can be “empty” or largely devoid of birds and mammals, where hunting pressure is high but land clearance and logging is low. Third, there is no established mechanistic basis through which changes to animal abundances and functions can be inferred based on changes to HANPP; at best, efforts to use HANPP as a proxy for the animal component of a biosphere integrity planetary boundary would rely on correlations. For all these reasons, we suggest that an energetics-based measure of animal-mediated ecosystem function would complement HANPP by revealing a different aspect of biosphere change.

To test our assumption that HANPP is *not* an accurate proxy for changes to animal-mediated ecosystem functions, we used a linear regression to test the strength of the relationship between proportional HANPP and the proportional intactness of energy flows through birds and mammals. We expected to find a weak correlation between the two metrics, indicating their correlation with aggregate human impacts on a land use, but expected the relationship to be noisy, given the different ways plants and animals respond to human activity. As expected, we found a weak, noisy relationship between increasing HANPP and declining energetic intactness of birds and mammals (slope = -0.24, p < 0.01, r^2^ = 0.15).

**
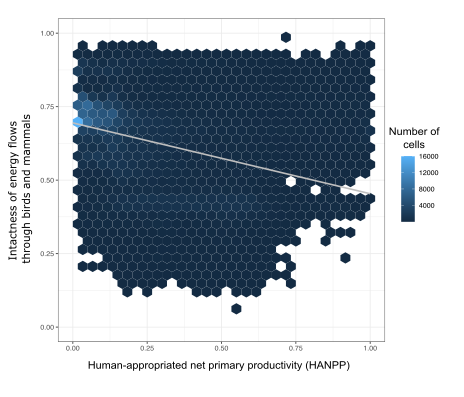
Figure S1. Relationship between HANPP and the intactness of energy flows through birds and mammals.** Across all 8x8 km cells in sub-Saharan Africa, proportional human appropriated net primary productivity (HANPP) was related to the proportional intactness of energy flows through birds and mammals.

We suggest the weakness of the relationships is not only a consequence of the different methodologies used to map HANPP and biodiversity intactness, but also reflects the divergent ways animal versus plant energetics respond to human land use. Factors such as overharvesting, protected area management, and competition with livestock are likely to affect animals and the functions they perform in ways that diverge from their impacts on plant primary productivity. We therefore conclude that mapping changes to energy flows through animal functional groups is likely to provide useful information about changes to the earth system not captured by changes to HANPP. Integrating HANPP and animal energetics provides a way forward for setting a planetary boundary for biosphere integrity that captures changes to both the plant and animal components of the biosphere.

**Supplementary data for the allometric equations used to calculate energy flows**

**Table S2**: Parameter values for the allometric equations for field metabolic rates (FMR) of mammals and birds, derived from ref.^6^.

| Animal Group | a | b | Mean log x | c | d | e | Equation number in ref.^1^ |
| --- | --- | --- | --- | --- | --- | --- | --- |
| Birds | 10.5 | 0.681 | 1.95 | 0.328 | 1.011 | 0.012 | 17: all birds |
| Mammals | 4.21 | 0.772 | 2.364 | 0.423 | 1.017 | 0.01 | 2: Eutherian mammals |

FMR = a* body mass ^ b, where the units are kJ day-1 for field metabolic rate and g for body mass.

Equation for calculating 95% confidence intervals of the prediction: 95%CIlog(FMR)= log(predicted FMR) ± c{d + e[log(body mass) – (mean log x)]^2}^0.5

**Table S3.** : Assimilation efficiencies (%) used for each feeding group. Uncertainties indicated are standard deviations across studies. Assimilation estimates for birds are by taxonomic order; the category ‘All birds’ is used for those orders for which data is not available. Derived from ref.^7^.

| **Diet** | **Vertebrates** | **Invertebrates** | **Fruit** | **Leaves** | **Seeds and Nuts** | **Nectar** |
| --- | --- | --- | --- | --- | --- | --- |
| **Struthioniformes** | 0.78±0.093 | 0.78±0.093 | 0.75±0.17 | 0.36±0.075 | 0.63±0.15 | 0.94±0.11 |
| **Gruiformes** | 0.34±0.093 | 0.34±0.093 | 0.45±0.17 | 0.59±0.016 | 0.63±0.14 | 0.94±0.11 |
| **Charadriiformes** | 0.69±0.058 | 0.69±0.058 | 0.75±0.17 | 0.44±0.15 | 0.63±0.14 | 0.94±0.11 |
| **Pelecaniformes** | 0.8±0.027 | 0.8±0.027 | 0.76±0.17 | 0.44±0.15 | 0.63±0.14 | 0.94±0.11 |
| **Strigiformes** | 0.77±0.09 | 0.77±0.09 | 0.75±0.17 | 0.44±0.15 | 0.63±0.14 | 0.94±0.11 |
| **Falconiformes** | 0.84±0.022 | 0.84±0.022 | 0.75±0.17 | 0.44±0.15 | 0.63±0.14 | 0.94±0.11 |
| **Accipitriformes** | 0.82±0.017 | 0.82±0.017 | 0.75±0.17 | 0.44±0.15 | 0.63±0.14 | 0.94±0.11 |
| **Ciconiiformes** | 0.8±0.032 | 0.8±0.032 | 0.75±0.17 | 0.44±0.15 | 0.63±0.14 | 0.94±0.11 |
| **Anseriformes** | 0.87±0.093 | 0.87±0.093 | 0.75±0.17 | 0.41±0.019 | 0.83±0.011 | 0.94±0.11 |
| **Galliformes** | 0.7±0.098 | 0.7±0.098 | 0.57±0.035 | 0.42±0.017 | 0.65±0.026 | 0.94±0.11 |
| **Coliiformes** | 0.78±0.093 | 0.78±0.093 | 0.56±0.025 | 0.44±0.15 | 0.63±0.14 | 0.94±0.11 |
| **Piciformes** | 0.64±0.093 | 0.64±0.093 | 0.75±0.17 | 0.61±0.024 | 0.63±0.14 | 0.94±0.11 |
| **Passeriformes** | 0.76±0.011 | 0.76±0.011 | 0.67±0.015 | 0.76±0.066 | 0.8±0.01 | 0.9±0.039 |
| **All birds** | 0.78±0.093 | 0.78±0.093 | 0.75±0.17 | 0.44±0.15 | 0.63±0.14 | 0.94±0.11 |

**Table S4.** : Assimilation efficiencies (%) used for each feeding group. Uncertainties indicated are standard deviations across studies. Assimilation estimates for mammals are by taxonomic group (for ruminants) and body size. Values for other are derived from ref.^8^. Values for large insectivores (> 0.5kg) come from refs.^9,10^. Values for *Proboscidea* come from ref.^11^. Values for *Perissodactyla* come from refs.^12,13^. Values for other ruminants come from ref.^14^. Values for *Hippopotamidae* come from ref.^15^ Values for *Suidae* come from ref.^16^.

| **Diet** | **Vertebrates** | **Invertebrates** | **Fruit** | **Leaves** | **Seeds and Nuts** | **Nectar** |
| --- | --- | --- | --- | --- | --- | --- |
| Other >0.5kg | 85±5.8 | 65±5.9 | 83±8.5 | 32±8.4 | 83±8.5 | 94±11 |
| Other <0.5kg | 85±5.8 | 88±5.9 | 83±8.5 | 46±10.7 | 83±8.5 | 94±11 |
| Perissodactyla |  |  |  | 43±8.4 |  |  |
| Suidae |  |  |  | 64±8.4 |  |  |
| Hippopotamidae |  |  |  | 54±8.4 |  |  |
| Proboscidea |  |  |  | 19±8.4 |  |  |
| Other ruminants |  |  |  | 50±8.4 |  |  |

**Reconstruction of historical large mammal ranges.**

Historical range maps are not available on the IUCN red list database for 11 large mammal species. We reconstructed range maps by adapting range maps from other sources, following ref.^1^. These range maps were than adjusted for area of habitat, following ref.^17^. We used the following sources for the relevant species: *Loxodonta africana*^1^. *Loxodonta cyclotis*^1^*, Ceratotherium simum*^1^*, Diceros bicornis*^1^*, Equus zebra*^1^*, Giraffa camelopardalis*^1^*, Equus quagga*^18^*, Equus grevyi*^18^*, Taurotragus derbianus*^18^*, Panthera leo*^19^*,* and *Lycaon pictus*^20^.

**Summary of Data and R Scripts for Calculating a Spatially Explicit Biodiversity Intactness Index (BII)**

The energetics analysis of sub-Saharan African birds and mammals was calculated in R using a the R script *Loft.Energetics.2407017.R*. The original data in the analysis come from a number of publicly available datasets. To ease code reproducibility, all the input data used in the analysis (which in some cases have been cleaned or filtered) are also uploaded here. The script in its entirety takes ~72 hours to run on a reasonably powerful laptop. To save the user time, some computationally intensive intermediate data products are provided in these files, although each of these can also be produced by running the script in its entirety. Further information on code structure is provided in the annotated script itself. Any questions and reasonable requests for additional data products should be directed to the corresponding author at [ty.loft@chch.ox.ac.uk](mailto:ty.loft@chch.ox.ac.uk). Below we list the annotated contents of this folder.

**Main Folder**

*Read Me Document (i.e. This document)*

- Readme_Energetics_RData

*R* *Script*

- Loft.Energetics.2407017

**Biodiversity Intactness Index Data (**data_bii_outputs**)**

*These data are used to estimate how species abundances change in response to land use change, according to the Biodiversity Intactness Index. They come from Clements et al., (2024) and Clements et al., (in review).* [*https://doi.org/10.1038/s41597-023-02832-6*](https://doi.org/10.1038/s41597-023-02832-6)

**Energetics Data (**data_energetics**)**

*These data are used to estimate the mean annual energy consumption (per unit area) of each African bird and mammal species. They include data on species traits, including body mass, diet composition, assimilation efficiency, and population density. The mammal trait data come from the Elton Traits database (*[*https://doi.org/10.1890/13-1917.1*](https://doi.org/10.1890/13-1917.1)*). The bird trait data come from the Avonet database (*[*https://doi.org/10.1111/ele.13898*](https://doi.org/10.1111/ele.13898)*). The data on assimilation efficiencies are based on the analysis in Malhi et al., (2022, Nature,* [*https://doi.org/10.1038/s41586-022-05523-1*](https://doi.org/10.1038/s41586-022-05523-1)*)*

**Species Range and Area of Habitat Data (**data_sp_range_habitat**)**

*These data are used to determine the bird and mammal species present in each 8x8 km cell in sub-Saharan Africa. They habitat-adjusted species range maps come from Beyer and Manica (2020,* [*https://doi.org/10.1038/s41467-020-19455-9*](https://doi.org/10.1038/s41467-020-19455-9)*). The raw range maps for mammals come from the IUCN (*[*https://www.iucnredlist.org/resources/spatial-data-download*](https://www.iucnredlist.org/resources/spatial-data-download)*) and the raw range maps for birds come from Birdlife International (*[*https://datazone.birdlife.org/species/requestdis*](https://datazone.birdlife.org/species/requestdis)*)*

**Intermediate Outputs**

*These data are intermediate outputs from the R Script, included to ease rapid reproducibility of key sections of the code.*

**Supplementary Data References**

1. Hempson, G. P., Archibald, S. & Bond, W. J. A continent-wide assessment of the form and intensity of large mammal herbivory in Africa. *Science* **350**, 1056–1061 (2015).

2. Richardson, K. *et al.* Earth beyond six of nine planetary boundaries. *Science Advances* **9**, eadh2458 (2023).

3. Haberl, H., Erb, K.-H. & Krausmann, F. Human Appropriation of Net Primary Production: Patterns, Trends, and Planetary Boundaries. *Annual Review of Environment and Resources* **39**, 363–391 (2014).

4. Newbold, T. *et al.* Ecological traits affect the response of tropical forest bird species to land-use intensity. *Proc. R. Soc. B.* **280**, 20122131 (2013).

5. Smit, I. P. J. & Prins, H. H. T. Predicting the Effects of Woody Encroachment on Mammal Communities, Grazing Biomass and Fire Frequency in African Savannas. *PLOS ONE* **10**, e0137857 (2015).

6. Nagy, K. A., Girard, I. A. & Brown, T. K. Energetics of Free-Ranging Mammals, Reptiles, and Birds. *Annual Review of Nutrition* **19**, 247–277 (1999).

7. Barlein, F. Energy and nutrient utilization efficiencies in birds--a review. in *Proceedings of the 22nd International Ornithological Congress* (eds. Adams, N. & Slotow, R.) (1999).

8. Crocker, D., Hart, A., Gurney, J. & McCoy, C. Estimating daily food intake of wild birds and mammals. (2002).

9. Panaino, W. *et al.* Do seasonal dietary shifts by Temminck’s pangolins compensate for winter resource scarcity in a semi-arid environment? *Journal of Arid Environments* **197**, 104676 (2022).

10. Williams, J. B., Anderson, M. D. & Richardson, P. R. K. SEASONAL DIFFERENCES IN FIELD METABOLISM, WATER REQUIREMENTS, AND FORAGING BEHAVIOR OF FREE-LIVING AARDWOLVES. *Ecology* **78**, 2588–2602 (1997).

11. Ruggiero, R. G. Seasonal forage utilization by elephants in central Africa. *African Journal of Ecology* **30**, 137–148 (1992).

12. Pozdnyakova, M. K., Zharkikh, T. L., Yasinetskaya, N. I. & Kolesnikov, M. P. Quantitative assessment of feeding in a free-ranging group of Przewalski horses, Equus przewalskii, in a steppe habitat (the Askania Nova Biosphere Reserve). *Biol Bull Russ Acad Sci* **38**, 726–734 (2011).

13. Abaturov, B. D., Kazmin, V. D. & Kolesnikov, M. P. Nutrition of bison (Bison bison), camels (Сamelus bactrianus), and horses (Equus caballus) from their joint grazing on an isolated steppe pasture. *Biol Bull Russ Acad Sci* **43**, 918–925 (2016).

14. Pellew, R. A. Food consumption and energy budgets of the giraffe. *Journal of Applied Ecology* 141–159 (1984).

15. Schwarm, A. *et al.* Digestion studies in captive Hippopotamidae: a group of large ungulates with an unusually low metabolic rate. *Journal of Animal Physiology and Animal Nutrition* **90**, 300–308 (2006).

16. Gallagher, J. F., Varner, L. W. & Grant, W. E. Nutrition of the Collared Peccary in South Texas. *The Journal of Wildlife Management* **48**, 749–761 (1984).

17. Beyer, R. M. & Manica, A. Historical and projected future range sizes of the world’s mammals, birds, and amphibians. *Nat Commun* **11**, 5633 (2020).

18. UCN/SSC Antelope Specialist Group. *African Antelope Database*. https://portals.iucn.org/library/efiles/documents/ssc-op-021.pdf (1998).

19. IUCN SSC Cat Specialist Group. *Conservation Strategy for the Lion in West and Central Africa*. https://www.cms.int/sites/default/files/document/IUCN_CatSG_2006_West_and_Central_Africa_Lion_Conservation_Strategy.pdf (2006).

20. Macdonald, D., Woodroffe, R. & Ginsberg, J. *Status Survey and Conservation Action Plan: The African Wild Dog*. https://portals.iucn.org/library/sites/library/files/documents/1997-052.pdf (1997).
